# Identifying perimenopause phenotypes among women living with and without HIV: A latent class analysis

**DOI:** 10.1093/geroni/igaf122.4328

**Published:** 2025-12-31

**Authors:** Maeve McNamara, Lauren Collins, C Christina Mehta, Anna Rubtsova, Camille Vaughan, Alexis Bender

**Affiliations:** Emory University, Atlanta, Georgia, United States; Emory University, Atlanta, Georgia, United States; Emory University, Atlanta, Georgia, United States; Emory University, Atlanta, Georgia, United States; Emory University, Atlanta, Georgia, United States; Emory University, Atlanta, Georgia, United States

## Abstract

There is increasing appreciation for the multidimensional impact that perimenopause symptoms have on women in mid-life. We sought to identify distinct classes of menopause symptomatology and whether these classes differ by HIV status and sociodemographic and clinical characteristics. We conducted a latent class analysis (LCA) of women with and without HIV in late perimenopause enrolled in the Women’s Interagency HIV Study who attended at least two study visits between 2007-2019. Late perimenopause was defined using STRAW +10 criteria. We used LCA to identify unique groups of participants based on the frequency of nine perimenopause symptoms (hot flashes, cold sweats, night sweats, musculoskeletal stiffness/soreness, irritability, nervousness, and three types of sleep disturbances). We assessed differences in class membership by HIV serostatus; sociodemographic and clinical characteristics, including non-AIDS comorbidity (NACM) burden, a measure of multimorbidity. Among 818 women (590 with HIV, 228 without HIV) aged 35-58 years (Median age 48), 67% identified as Black, and 9.3% reported hormonal birth control use. Age and symptom frequency did not differ by HIV serostatus. LCA identified five symptom classes: (1) sore, tired, and on edge, (2) hot, sore, and tired, (3) tired, (4) hot, sore, and irritable, and (5) overall low symptoms. Class membership significantly differed by depressive symptoms, age, hormonal birth control use, NACM burden, alcohol use, and income, but not by HIV serostatus, race, tobacco use, or BMI. We identified heterogeneity in perimenopause symptomology regardless of HIV. These data may inform provider education to improve patient validation and agency in seeking perimenopausal care.

